# Preliminary Insights from a Multi‐Ancestry TWAS in Alzheimer’s Disease in African and European Populations

**DOI:** 10.1002/alz.092593

**Published:** 2025-01-03

**Authors:** Xinyu Sun, Makaela Mews, Nicholas R. Wheeler, Penelope Benchek, Christiane Reitz, Adam C. Naj, Jennifer Elizabeth Below, Brian W. Kunkle, Anthony J. Griswold, William S. Bush

**Affiliations:** ^1^ Department of Population and Quantitative Health Sciences, Case Western Reserve University, Cleveland, OH USA; ^2^ System Biology & Bioinformatics; Department of Nutrition; Case Western Reserve University, Cleveland, OH USA; ^3^ Case Western Reserve University School of Medicine, Cleveland, OH USA; ^4^ Case Western Reserve University, Cleveland, OH USA; ^5^ Gertrude H. Sergievsky Center, Taub Institute for Research on the Aging Brain, Departments of Neurology, Psychiatry, and Epidemiology, College of Physicians and Surgeons, Columbia University, New York, NY USA; ^6^ Department of Biostatistics, Epidemiology, and Informatics, University of Pennsylvania Perelman School of Medicine, Philadelphia, PA USA; ^7^ Vanderbilt Genetics Institute and Division of Genetic Medicine, Vanderbilt University Medical Center, Nashville, TN USA; ^8^ John P. Hussman Institute for Human Genomics, Miller School of Medicine, University of Miami, Miami, FL USA; ^9^ John P. Hussman Institute for Human Genomics, University of Miami Miller School of Medicine, Miami, FL, USA, Miami, FL USA; ^10^ Dr. John T. Macdonald Foundation Department of Human Genetics, University of Miami Miller School of Medicine, Miami, FL USA; ^11^ Department of Population and Quantitative Health Sciences, Institute for Computational Biology, Case Western Reserve University, Cleveland, OH USA

## Abstract

**Background:**

Recent advances in Alzheimer’s Disease (AD) research have emphasized the importance of recruiting from diverse populations. Notably, African‐descent individuals have an almost doubled risk of developing AD compared to European‐descent individuals. Transcriptome‐wide association studies (TWAS) have advanced the analysis of non‐coding variants by integrating gene expression with GWAS data. The newly developed Multi‐ancEstry TRanscriptOme‐wide analysis (METRO) method leverages gene expression data from multiple ancestries, enhancing TWAS power. Addressing this knowledge gap, our study utilized METRO, incorporating both African and European expression and genotype data from the Multi‐Ancestry Genomics, Epigenomics, and Transcriptomics of Alzheimer’s (MAGENTA), to identify AD‐associated genes in each population.

**Method:**

Quality control (QC) on genotype data included filtering SNPs with MAF>0.05 and removing samples with outlier global principal components (PCs). Post‐QC, 199 African and 236 European samples from MAGENTA, with both whole‐blood expression and genotype data, were retained. cis‐eQTL analysis was performed for each protein‐coding gene within a 500kb region, adjusting for age, sex, PCs, and PEER factors. We obtained African American AD GWAS statistics from Kunkle et al. 2021 (n = 8,006) and European AD GWAS statistics from Douglas et al. 2021, excluding 23andMe and UKBB samples (n = 398,058). High‐coverage 1kGP WGS data served as the linkage disequilibrium (LD) reference panel for GWAS statistics. METRO analysis utilized both ancestries’ summary‐level eQTL and GWAS statistics.

**Result:**

We identified 276 statistically significant genes using European‐descent individuals after Bonferroni correction. In contrast, no significant genes were found using African‐descent individuals, except within the *APOE* locus. Excluding *APOE* locus genes, gene‐set enrichment analysis using DisGeNET revealed enrichment in AD and related dementia pathways among the top 10 pathways. Among the 246 significant genes, excluding *APOE* locus, identified in individuals of European‐descent, 131 exhibited the same gene‐trait association direction in individuals of African‐descent.

**Conclusion:**

The multi‐ancestry TWAS identified 276 genes potentially associated with AD, particularly in pathways related to the disease. These findings may enhance our understanding of AD genetic risk. The limited sample size in African‐descent AD GWAS may explain the lack of significant gene identification beyond the *APOE* locus. Future studies with more diverse populations are essential for discovering novel AD‐related genes in underrepresented groups.

